# Participant Informed Consent in Cluster Randomized Trials: Review

**DOI:** 10.1371/journal.pone.0040436

**Published:** 2012-07-06

**Authors:** Bruno Giraudeau, Agnès Caille, Amélie Le Gouge, Philippe Ravaud

**Affiliations:** 1 INSERM, U738, Paris, France; 2 INSERM, CIC 202, Tours, France; 3 Université François Rabelais, Tours, France; 4 CHRU de Tours, Tours, France; 5 Assistance Publique–Hôpitaux de Paris, Hôpital Hôtel Dieu, Centre d'Epidémiologie Clinique, Paris, France; 6 Université Paris Descartes Sorbonne Paris Cité, Paris, France; University of Buea, Cameroon

## Abstract

**Background:**

The Nuremberg code defines the general ethical framework of medical research with participant consent as its cornerstone. In cluster randomized trials (CRT), obtaining participant informed consent raises logistic and methodologic concerns. First, with randomization of large clusters such as geographical areas, obtaining individual informed consent may be impossible. Second, participants in randomized clusters cannot avoid certain interventions, which implies that participant informed consent refers only to data collection, not administration of an intervention. Third, complete participant information may be a source of selection bias, which then raises methodological concerns. We assessed whether participant informed consent was required in such trials, which type of consent was required, and whether the trial was at risk of selection bias because of the very nature of participant information.

**Methods and Findings:**

We systematically reviewed all reports of CRT published in MEDLINE in 2008 and surveyed corresponding authors regarding the nature of the informed consent and the process of participant inclusion. We identified 173 reports and obtained an answer from 113 authors (65.3%). In total, 23.7% of the reports lacked information on ethics committee approval or participant consent, 53.1% of authors declared that participant consent was for data collection only and 58.5% that the group allocation was not specified for participants. The process of recruitment (chronology of participant recruitment with regard to cluster randomization) was rarely reported, and we estimated that only 56.6% of the trials were free of potential selection bias.

**Conclusions:**

For CRTs, the reporting of ethics committee approval and participant informed consent is less than optimal. Reports should describe whether participants consented for administration of an intervention and/or data collection. Finally, the process of participant recruitment should be fully described (namely, whether participants were informed of the allocation group before being recruited) for a better appraisal of the risk of selection bias.

## Introduction

In a cluster randomized trial (CRT) “*intact social units, or clusters of individuals, rather than individuals themselves, are randomized*” [1]. Such a design is well adapted to assess organizational and behavioral interventions or health promotion programs, interventions that are usually implemented at the level of health organization units or geographical areas [2]. As well, randomization of clusters rather than individuals may prevent contamination (i.e., control participants may adopt the experimental intervention) [3] and is therefore also used to assess interventions at the patient level such as therapeutic or preventive education programs. In addition, cluster randomization is often used to assess interventions aimed at curing or preventing transmission of contagious diseases, which allows for assessing both the direct effect of an intervention and its indirect effect due to the impact of the intervention on the transmission of the disease [4], [5]. The design is now considered well adapted for pragmatic trials [6] – and also named “real-world trials” [7] – as “*a way to allow for real world practice within study centers while addressing intercenter bias by randomizing those to the study interventions.*” The use of cluster randomization has greatly increased over the past 20 years [8] and even motivated an extension of the CONSORT statement [9].

Ethical issues associated with medical research are based on the Nuremberg code [10]. The first of the 10 criteria of the code relates to subjects' consent to participate in a study: “*the voluntary consent of the human subject is absolutely essential*.” The Declaration of Helsinki [11] is the embodiment of the Nuremberg code. Of note, its 1996 version described situations in which informed consent is not obtained: “*if the physician considers it essential not to obtain informed consent, the specific reasons for this proposal should be stated in the experimental protocol for transmission to the independent committee*,” but in the current version (Tokyo, 2004), this notion no longer appears. Finally, the Council for International Organizations of Medical Sciences (CIOMS) recently updated its international ethical guidelines for epidemiological studies [12], and one section is devoted to CRTs. The guidelines state that “*waiver of informed consent is to be regarded as uncommon and exceptional, and must in all cases be approved by an ethical review committee*.”

CRTs raise at least 4 concerns for handling participant informed consent [13], [14], [15], [16]. First, the hierarchical structure of such trials implies the consideration of 2 levels of consent. The first level is the “*guardian*,” as defined by Edwards *et al* [13], also named “*gatekeeper*” by Hutton [14] or the “*cluster representation mechanism*” by the Medical Research Council [15], who must agree to participation and randomization. The other level is participants embedded within clusters. Second, some interventions (such as fluoridation of water supply or computer-based tools to help physicians while prescribing) apply (or not) to the whole cluster, and individual participants have no opt-out option [12], [15]. Participant consent can therefore cover different things, and thus, Hutton [14] distinguished 3 types of consent: (i) consent that routinely held data on individuals be collected, (ii) consent regarding the collection of supplementary data and (iii) consent for active participation. Third, randomizing large clusters such as hospitals, villages, or geographical areas implies logistic difficulties that cannot be overcome to obtain individual informed consent [17]. Fourth, full information given to the cluster participant may compromise the internal validity of the trial because of selection bias (lack of allocation concealment induced by a randomization of clusters before recruitment of participants) [18], [19] and group contamination [13], [15], [20]. Blinding participants to the study hypothesis or delivering differential information [21], [22] may then greatly help prevent or reduce bias. These situations led the CIOMS to consider the possibility of a transfer of consent from the individual to the cluster level: the person in charge of the cluster “*has authority to give permission for the cluster to participate in the study and to be assigned on a random basis to one arm or another of the study*,” and thus, consent to the study is collective [12]. In addition, reporting of research ethics review and informed consent has recently been shown to be inadequate, with an injunction for authors to report, in addition to research ethics approval, “*whether informed consent was sought, from whom consent was sought, and what consent was for*” [23].

We thus performed a systematic review of recently published reports of CRTs, completed by surveying the corresponding authors of selected reports. We aimed to assess whether participant-informed consent was required, the nature of the consent (i.e., what participants gave consent for), and whether partial information had been delivered to included participants to help prevent bias.

## Methods

### Search strategy

We searched MEDLINE via PubMed using the following syntax: (cluster OR clustered OR group-randomized OR group-randomised OR community-randomized OR community-randomised) AND “clinical trial”[Publication Type] AND (2008[Publication Date]). Two of us (BG, ALG) independently screened the titles and abstracts of retrieved reports. Any discordance was resolved by consensus.

### Selection of relevant reports

We included all articles reporting primary or secondary analyses of CRTs. For secondary analyses, we retrieved the articles reporting the primary results. We discarded articles reporting trial protocols, because we had no opportunity to check compliance with information and consent issues.

### Assessment of selected reports

We generated a standardized data collection form that was pilot-tested and agreed upon by the 3 reviewers (BG, AC, ALG). Then, rotating pairs of reviewers independently abstracted information from sets of 30 of the selected articles. After data from each set had been abstracted, discrepancies were resolved by consensus.

### General characteristics of selected reports

We recorded the settings (countries with high- or low/middle-income economies based on the World Bank classification [http://data.worldbank.org/node/8]), the medical field, the randomization unit and the kind of intervention. Using the Eldridge *et al* typology of intervention [20], we classified trials according to whether the intervention applied at the level of the individual or the cluster. We thus considered that the “individual-cluster” type, as defined by Eldridge *et al*, referred to interventions that apply at the level of the individual, whereas the 3 other types (“professional-cluster”, “external-cluster” and “cluster-cluster”) referred to interventions that apply at the level of the cluster. Moreover, because the Eldridge *et al* types are not exclusive, a complex intervention may imply both individual- and cluster-level interventions. Such trials were classified in the cluster-level group.

### Reporting on ethics committee, participant information and consent

We checked whether ethics committee approval was reported. We also recorded whether the handling of participant consent was reported: whether individual participant consent was obtained or not required and whether the consent was verbal or written. We further collected whether participant information was reported as partial or differential.

### Author survey

A questionnaire was sent to all corresponding authors with a current email address. The initial invitation was followed by 2 reminders.

The survey asked authors about complementary information, including how participants were selected (i.e., whether recruitment was before cluster randomization and by an independent recruiter). It also asked whether the group allocation was specified, in case participants were recruited after clusters had been randomized. Regarding participant consent, the survey queried the type of consent as defined by Hutton [14] (i.e. (i) consent that routinely held data on individuals be collected, (ii) consent regarding the collection of supplementary data and (iii) consent for active participation), and we then assessed the proportion of trials for which consent was for data collection only (i.e. the first 2 situations considered by Hutton). Authors were also asked about the existence of cluster guardians [13] and whether guardians had authority to consent for whole clusters. The survey asked whether their trial contained an opt-out option (i.e., whether participants could avoid the intervention or not) [15]. Finally, authors had the opportunity to express their perception of the management of participant-informed consent in CRTs.

### Complete participant information and selection bias risk

Most CRTs are non-blinded trials. Allocation concealment is then compromised anytime full information is delivered to participants by a non-blinded recruiter. We therefore used the complementary information provided by authors to identify the potential risk of selection bias due to both the participant selection process used and the completeness of delivered information.

### Statistical analysis

Data are reported as number (%). Percentages were compared by chi-square tests or Fisher exact tests, when necessary. Statistical analyses involved use of SAS 9.2 (SAS Inst., Cary, NC, USA).

## Results

**Figure 1:**
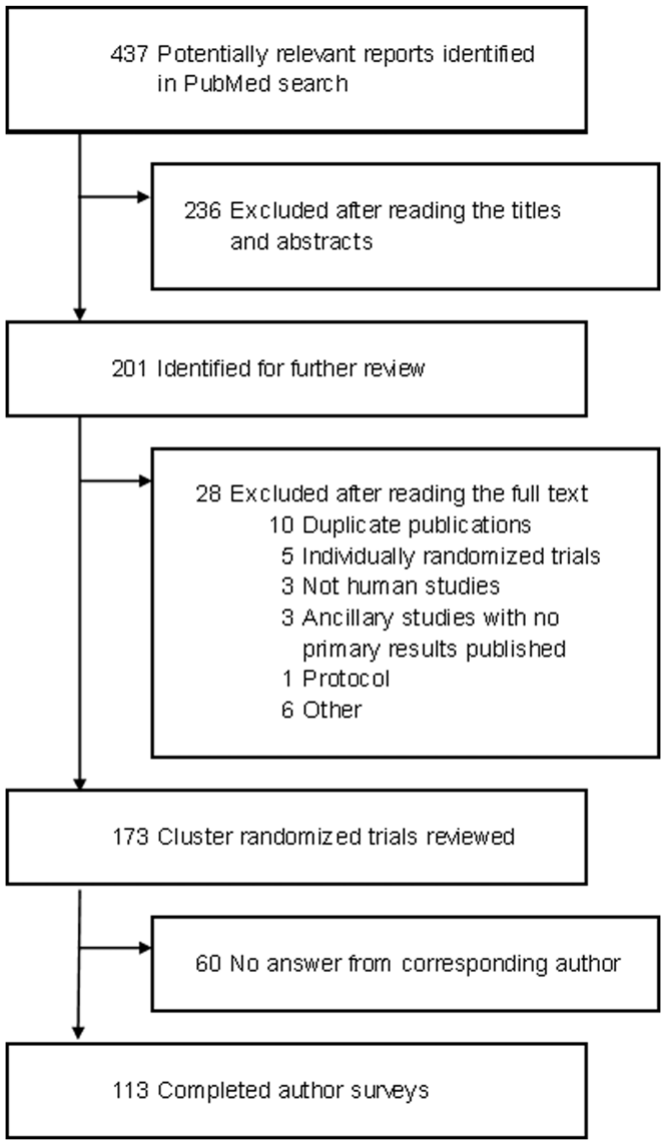
Study selection.

The search strategy generated 437 reports: 173 were eligible and were appraised (Figure 1). Of them, 18 were actually secondary analyses of previously published CRTs. The median publication date [quartiles] for these 18 reports was 2006 [2005; 2007].

### Systematic review results

#### Characteristics of selected studies

Table 1 details the general characteristics of the selected articles. About one-quarter of the trials took place in countries with low- or middle-income economies. Randomization units were mostly practices or health professionals (25.4%), villages or community/geographical areas (20.8%) or schools/classrooms (15.0%). The intervention was applied at the individual level in 67 trials (38.7%), whereas in the remaining 106 (61.3%), at least some part of the intervention was applied at the cluster level.

**Table 1:** Systematic review of ethics committee approval and participant information and consent in reports of cluster randomized trials – characteristics of trials.

	Selected reports (n = 173)	Reports for which authors answered the survey (n = 113)
**Country setting** a		
High-income economy	130 (75.1)	93 (82.3)
Low- or middle-income economy	43 (24.9)	20 (17.7)
**Medical field** b		
Prevention/health promotion	102 (59.0)	64 (56.6)
Diagnostics/screening	12 (6.9)	7 (6.2)
Therapeutic	41 (23.7)	25 (22.1)
Quality of care	46 (26.6)	33 (29.2)
Other	2 (1.2)	0
**Randomization unit (i.e., cluster)** c		
Hospital	9 (5.2)	7 (6.2)
Ward	10 (5.8)	6 (5.3)
Health center	14 (8.1)	9 (8.0)
Residential care home	7 (4.0)	5 (4.4)
Practice or health professional	44 (25.4)	34 (30.1)
School/classroom	26 (15.0)	19 (16.8)
Family/household	2 (1.2)	2 (1.8)
Village or community/geographical area	36 (20.8)	14 (12.4)
Other	26 (15.0)	18 (15.9)
**Kind of intervention** b		
Behavioral intervention (e.g., dietary or activity program)	63 (36.4)	46 (40.7)
Therapeutic education (e.g., leaflets or booklets)	9 (5.2)	6 (5.3)
Device for the participant (e.g., hip pad)	16 (9.2)	12 (10.6)
Device/tool provide to the physician (e.g., computer-based tool)	18 (10.4)	12 (10.6)
Pharmacological treatment, supplementation, vaccine	20 (11.6)	7 (6.2)
Health professional activity in consultation (e.g., training for health professionals, guidelines)	64 (37.0)	44 (38.9)
Additional staff (e.g., liaison nurses)	24 (13.9)	16 (14.2)
Feedback to health professionals	10 (5.8)	6 (5.3)
Cluster-wide information (e.g., information campaign for screening)	7 (4.0)	2 (1.8)
Other	23 (13.3)	16 (14.2)
**Eldridge's classification of intervention** b		
Individual cluster (e.g., treatment/information given to patients)	102 (59.0)	66 (58.4)
Professional cluster (e.g., guidelines, training for physicians)	84 (48.5)	59 (52.2)
External cluster (e.g., additional staff)	22 (12.7)	14 (12.4)
Cluster cluster (e.g., change in organization, cluster-wide information)	13 (7.5)	5 (4.4)
**Level of intervention**		
Individual	67 (38.7)	41 (36.3)
Cluster	106 (61.3)	72 (63.7)

Data are numbers (percentages).

a World Bank classification (http://data.worldbank.org/node/8).

b Non-exclusive classification.

c In one report, data for 2 trials were reported, with both hospitals and practices as randomization units.

#### Ethics committee approval, participant information and consent

Most reports (89.6%) mentioned ethics committee approval (Table 2). Only one report explicitly stated that no committee was contacted. In this trial, United Kingdom hospitals were randomized to assess the effect of education or information on breast-care nurses' knowledge. The report stated that “*formal approval from a local research ethics committee was not required because study participants were professional nurses*.”

**Table 2:** Systematic review of ethics committee approval and participant information and consent in reports of cluster randomized trials – reporting of ethics committee approval and participant information and consent.

	Selected reports			Reports for which authors answered the survey		
	Total (n = 173)	Level of intervention		Total (n = 113)	Level of intervention	
		Individual (n = 67)	Cluster (n = 106)		Individual (n = 41)	Cluster (n = 72)
**Ethics committee approval**						
Obtained	155 (89.6)	61 (91.0)	94 (88.7)	103 (91.1)	37 (90.2)	66 (91.7)
No committee contacted	1 (0.6)	1 (1.5)	0	1 (0.9)	1 (2.4)	0
Unclear	17 (9.8)	5 (7.5)	12 (11.3)	9 (8.0)	3 (7.3)	6 (8.3)
**Informed participant consent to participate**						
Obtained	134 (77.5)	63 (94.0)	71 (67.0)	90 (79.6)	39 (95.1)	51 (70.8)
No consent required	9 (5.2)	1 (1.5)	8 (7.5)	6 (5.7)	1 (2.4)	5 (6.9)
Not specified	30 (17.3)	3 (4.5)	27 (25.5)	17 (15.0)	1 (2.4)	16 (22.2)
**Consent form, if obtained**						
Written	86 (64.2)	41 (65.1)	45 (63.4)	59 (65.6)	24 (61.5)	35 (68.6)
Verbal	11 (8.2)	7 (11.1)	4 (5.6)	2 (2.2)	1 (2.6)	1 (2.0)
Not specified	37 (27.6)	15 (23.8)	22 (31.0)	29 (32.2)	14 (35.9)	15 (29.4)
**Items combined: ethics committee approval & informed participant consent**						
Ethics committee approval and informed participant consent to participate	124 (71.7)	60 (89.5)	64 (60.4)	83 (73.5)	37 (90.2)	46 (63.9)
Ethics committee approval and no consent required	7 (4.0)	0	7 (6.6)	5 (4.4)	0	5 (6.9)
No ethics committee contacted – no consent	1 (0.6)	1 (1.5)	0	1 (0.9)	1 (2.5)	0
≥1 piece of information missing	41 (23.7)	6 (9.0)	35 (33.0)	24 (21.2)	3 (7.3)	21 (29.2)
**Characteristics of participant information** a						
Partial information	8 (6.0)	5 (7.9)	3 (4.2)	6 (6.7)	3 (7.7)	3 (5.9)
Differential information	3 (2.2)	2 (3.2)	1 (1.4)	3 (3.3)	2 (5.1)	1 (2.0)
Not specified	123 (91.8)	56 (88.9)	67 (94.4)	81 (90.0)	34 (87.2)	47 (92.1)

Data are number (%);

a For reports stating an informed consent was obtained.

Most reports (134, 77.5%) stated that participant consent was obtained, with a higher proportion in individual-level intervention trials (94.0%) than in cluster-level intervention trials (67.0%, p<0.001). Otherwise, 9 reports (5.2%) explicitly stated that informed consent was not required. In 7 of these reports, the intervention referred to health professional activity in consultation (such as guidelines, workshops, electronic devices); in 1, the intervention consisted of an educational program provided in classrooms; and the last one was the report for which the authors stated they did not contact any ethics committee (cf supra). Of note, 7 of these trials took place in countries with high-income economies. In 11 reports, participant consent was declared to be oral: 10 took place in countries with low- or middle-income economies and 1 in the United Kingdom, assessing the impact of a rapid PCR-based screening test for methicillin-resistant *Staphylococcus aureus* infection.

Thus, for about 1 in 4 reports (23.7%), ethical issues management was not fully reported (i.e., ethics committee approval was not stated and/or no information was provided as to whether informed consent was obtained or not required). This proportion was higher in cluster-level intervention trials (33.0%) than individual-level intervention trials (9.0%, p<0.001).

In 8 reports (6.0%), information given to participants was partial, and in 3 (2.2%), it was described as differential because participants from the control group were not told the nature of the intervention assessed. For 5 of these 11 reports, the assessed intervention referred to physician or nurse activity, and for 6, it was educational programs displayed at school, for sports teams or geographical areas.

### Author survey results

#### Characteristics of the sub-sample of studies with author responses

Of the 173 corresponding authors contacted, 113 (65.3%) answered the survey. The sub-sample of reports for which we obtained an author answer to our survey differed from the sub-sample of 60 reports for which we did not obtain an author answer. Trials more often took place in high-income than other economy countries (82.3% vs 61.7%, p = 0.03), with less often a pharmacological than other intervention (6.2% vs 21.7%) and less often a cluster–cluster intervention than other intervention (4.4% vs 13.3%, p = 0.03).

#### Existence of an opt-out option

In total, 52 authors (48.6%) considered that participants were not able to avoid the assessed intervention, and 49 (45.8%) acknowledged an opt-out option; 6 others (5.6%) responded “did not know.” The proportion of trials without an opt-out option was significantly higher for cluster- than individual-level trials (60.0% vs. 36.1%, p = 0.021). For the latter trials, most involved educational programs administered in schools or classrooms. The authors considered these interventions unavoidable, which means that providing consent for administration of an intervention was not possible. In such situations, authors then declared that participants consented for data collection only (cf infra).

#### Ethics committee approval, participant information and consent (Table 3)

Among the 113 completed surveys, 8 authors (7.3%) declared having difficulties in obtaining ethics committee approval. One mentioned difficulties in relation to the design: “*we first aimed to have an opt-out approach where participants would have been informed of the trial and invited to opt out if preferred. However, we ultimately chose a more standard opt-in consent.*” Another author specified that “*the ethics committee decided that the intervention part was development work, not needing a statement. The statement was obtained only for the data collection part*.” For the 6 other trials, difficulties were not related to design, or authors did not provide complementary information. Of note, one author who stated having ethics committee approval without difficulty later commented that it was difficult “*establishing [that] informed consent from patients was not required.*”

**Table 3:** Systematic review of ethics committee approval and participant information and consent in reports of cluster randomized trials – author survey of handling participant information and consent.

	Total (n = 113)		Level of intervention			
			Individual (n = 41)			Cluster (n = 72)
**Difficulties obtaining ethics committee approval**						
Yes	8 (7.3)		1 (2.4)			7 (10.1)
No	97 (88.2)		38 (92.7)			59 (85.5)
Not applicable (e.g., no ethics committee contacted)	4 (3.6)		2 (4.9)			2 (2.9)
Do not know	1 (0.9)		0			1 (1.4)
**Participant consent**						
Yes	98 (88.3)		39 (95.1)			59 (84.3)
No consent	13 (11.7)		2 (4.9)			11 (15.7)
**Consent form, if participant consented**						
Written	83 (89.2)		33 (89.2)			50 (89.3)
Verbal	10 (10.8)		4 (10.8)			6 (10.7)
**Type of consent, if participant consented**						
Consent for data collection only	43 (53.1)		28 (60.9)			15 (42.9)
**Items combined: ethics committee approval, participant consent and type of consent** a						
Ethics committee approval and participant consent for administration of an intervention	37 (33.9)		19 (46.3)			18 (26.5)
Ethics committee approval and participant consent for only collection of data (either routinely or additional)	41 (37.6)		15 (36.6)			26 (38.2)
Ethics committee approval and participant consent, without specification of the type of consent	15 (13.8)		4 (9.8)			11 (16.2)
Ethics committee approval and no participant consent	12 (11.0)		1 (2.4)			11 (16.2)
No ethics committee contacted	4 (3.7)		2 (4.9)			2 (2.9)
**Participant information**						
Yes	100 (90.9)		41 (100.0)			59 (85.5)
No information	10 (9.1)		0			10 (14.5)
**Communication mode, if participants were informed**						
**Nature of communication**						
Oral only	3 (3.0)		2 (4.9)			1 (1.7)
Oral and written	84 (84.0)		34 (82.9)			50 (84.7)
Written only	13 (13.0)		5 (12.2)			8 (13.6)
**How delivered**						
To each participant individually	62 (62.0)		20 (48.8)			42 (71.2)
To groups of participants	17 (17.0)		10 (24.4)			7 (11.9)
Both	21 (21.0)		11 (26.8)			10 (16.9)
**Characteristics of participant information**						
Partial information b	19 (19.0)		8 (19.5)			11 (18.6)
Differential information	18 (18.0)		5 (12.2)			13 (22.0)
**Participant allocation concealment: group allocation specified, if participants were informed and recruited after clusters had been randomized** c						
Yes		16 (30.2)		5 (25.0)	11 (33.3)	
Not specified		31 (58.5)		11 (55.0)	20 (60.6)	
Do not know		6 (11.3)		4 (20.0)	2 (6.1)	

Data are number (%).

a An ethics committee approval was considered obtained any time the corresponding author specified having difficulties or not in obtaining its approval.

b Information was considered as partial when (i) the study hypothesis was not specified or (ii) the nature of the control group and experimental group was not specified or (iii) the arm to which the participant would be allocated to was not specified (only for trials for which randomization of clusters took place before inclusion of participant).

c Participant inclusion was declared after cluster randomization in 66 trials. The corresponding authors of 53 of those reports answered this question.

Thirteen authors (11.7%) declared that participant consent was not required in their study, with no significant difference between cluster- and individual-level trials (15.7% vs. 4.9%, p = 0.127). For trials in which participant consent was required, for more than half (43/81, 53.1%), participants were asked to consent for data collection only. In these trials, assessed interventions were largely interventions involving physician or nurse professional activity (23 reports) or educational interventions implemented in schools/classrooms, daycare centers, sport teams or health centers (15 reports).

In an individually randomized trial, trialists have to obtain ethics committee approval and participant informed consent to receive an intervention. For our CRTs, only 37 authors (33.9%) declared that this complete process was fully respected. This proportion was higher for individual- than cluster-level trials (46.3% vs. 26.5%, p = 0.034).

Ten (9.1%) corresponding authors declared that no information was provided to participants, and in all cases, the trials involved a cluster-level intervention. Otherwise, among trials for which authors declared that participant recruitment occurred after randomization (i.e., a subsample of 66 trials), 31 of 53 authors (58.5%) declared that group allocation was not specified for participants. Proportions were similar among cluster- and individual-level trials.

#### Cluster guardian and intervention avoidability

In total, 60 authors (55.0%) stated the existence of cluster guardians in their trials (Table 4), and for 73.3%, the guardians were asked to give written consent (56.0% vs. 85.7% for trials with individual- vs. cluster-level interventions, p = 0.010). Of note, only 31 of the 60 authors (51.7%) considered whether cluster guardians had the authority to consent for the whole cluster, and opinions were balanced (16 vs. 15). We did not observe any relation with the nature of the randomization unit.

**Table 4:** Systematic review of ethics committee approval and participant information and consent in reports of cluster randomized trials – author survey of cluster guardians.

	Total (n = 113)	Level of intervention	
		Individual (n = 41)	Cluster (n = 72)
**Existence of a cluster guardian**			
Yes	60 (55.0)	25 (61.0)	35 (51.5)
No	49 (45.0)	16 (39.0)	33 (48.5)
**Written consent from guardian**			
Yes	44 (73.3)	14 (56.0)	30 (85.7)
No consent	15 (25.0)	10 (40.0)	5 (14.3)
Do not know	1 (1.7)	1 (4.0)	0
**Guardian has the authority to consent for the whole cluster**			
Yes	16 (47.1)	5 (35.7)	11 (55.0)
No	15 (44.1)	7 (50.0)	8 (40.0)
Do not know	3 (8.8)	2 (14.3)	1 (5.0)

Data are number (%).

### Participant information and selection bias

**Figure 2:**
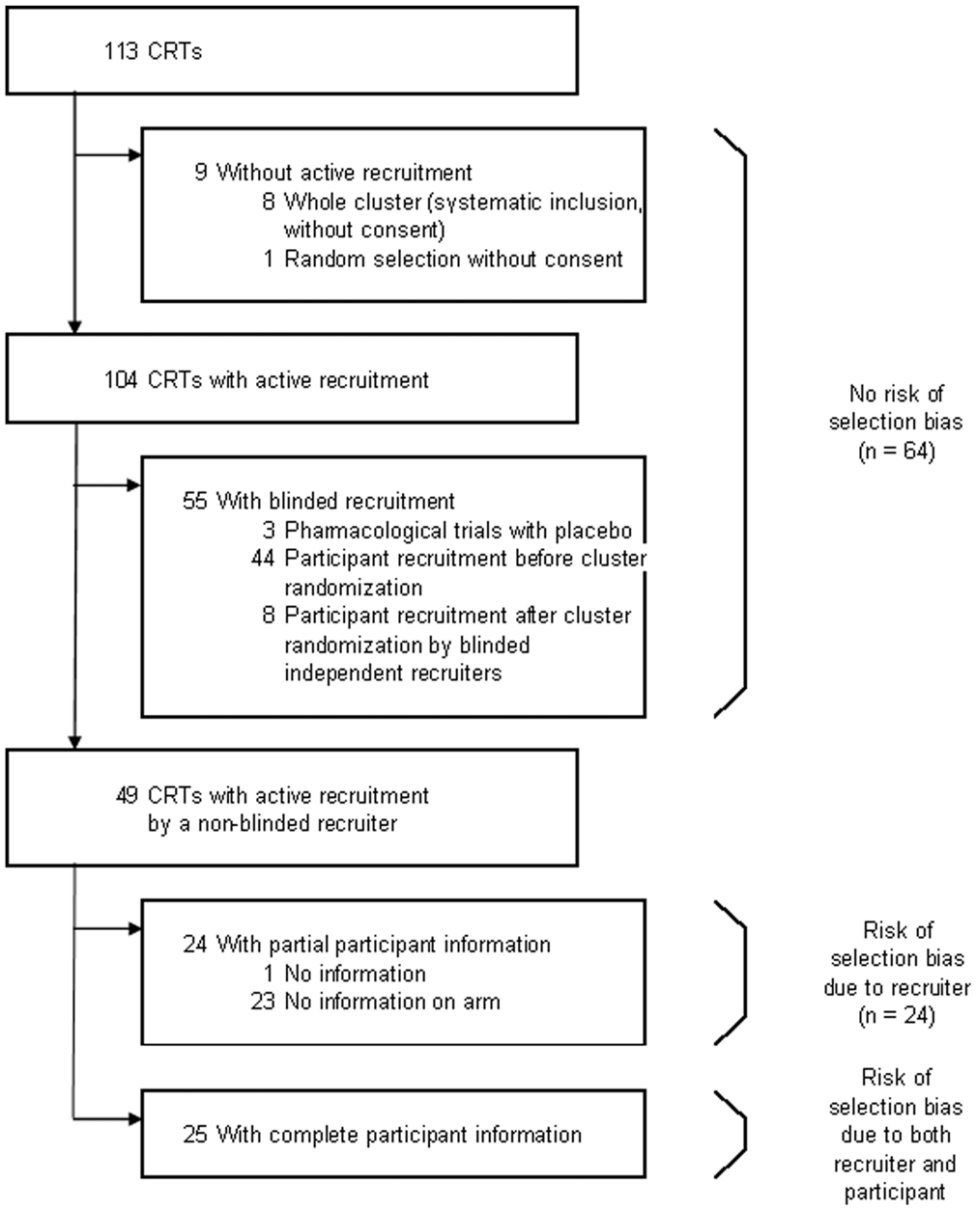
Risk of selection bias.

Figure 2 classifies the 113 trials according to the process used for participant selection and the risk of selection bias due to lack of allocation concealment. In total, 64 trials (56.6%) did not exhibit selection bias risk, because all participants within randomized clusters were systematically included and participants did not consent (i.e., there was no recruitment process) or because participant recruitment was blinded. Ways to blind recruitment included use of a placebo in a pharmacological trial (3 trials), participant recruitment before randomization of clusters (44 trials) (as advised by Puffer *et al* [24]), or blinded recruiters (8 trials).

For 24 trials (21.2%), the recruiter was not blinded, but participants were not fully informed. These situations are considered intermediate, because selection bias may be induced by a recruiter (who may select eligible participants), but the participant decision to be included is independent of the allocation result. Finally, for 25 trials (22.1%), both recruiters and participants were aware of the allocation results, with therefore a major risk of selection bias.

### Reporting (Table 5)

**Table 5:** Systematic review of ethics committee approval and participant information and consent in reports of cluster randomized trials – discrepancies between author answers and reporting.

Author statements	Numbers	Information stated in reports a
**Ethics committee approval**		
Obtained b	105	98 (93.3)
No ethic committee contacted	4	1 (25.0)
**Participant consent**		
Obtained	98	87 (88.8)
No consent	13	4 (30.8)
**Specifics of participant information**		
No information	10	3 (30.0)
Group allocation not specified c	31	4 (12.9)
**Specifics of participant recruitment**		
Recruitment before cluster randomization d	44	9 (20.5)
Blinded independent recruiter d	8	1 (12.5)

Data are number (%).

a Percentages represent the proportion of published reports stating the piece of information given by corresponding authors.

b An ethics committee approval was considered obtained any time the corresponding author specified having difficulties or not in obtaining its approval.

c Subset of trials for which authors answered that participants had been informed, and included after clusters had been randomized.

d Subset of trials for which we considered active recruitment took place.

**Table 6:** Authors' perception of management of participant informed consent in cluster randomized trials.

**Different levels of informed consent**
“*In cluster randomized trials, there are different levels of consent; you need to obtain consent at all levels for your study to be successful.*”
“*Very hard to get all the steps: school approval, family approval and children [who] desire to participate.*”
“*We implemented 3 layers of consent: cluster, clinical director; intervention, at the level of the clinician using a decision support tool and outcome; [and] the woman receiving care.*”
“*It is always at least a 2-step process.*”
**What do participants consent to?**
“*Participants do not consent to the intervention as such but to the follow-up procedures.*”
“*(…) communicating to participants exactly what they were consenting to is complex.*”
“*(…) all intervention children receive the program but only those who … consent for the assessment components provide data.*”
**Should participant information be complete? Risk of selection bias due to lack of allocation concealment**
“*Do we have the right not to inform completely some patients because they belong to one cluster? The implications [are for] partial information. This of course depends on the type of intervention and the risk/benefits of the compared strategies.*”
“*The main problem is contamination across groups if there were to be full transparency.*”
“*Asking for individual consent after cluster consent and allocation does increase the potential for selection bias.*”
**Can participants avoid the intervention?**
“*Difficult in case of interventions that have to be applied to all patients at the time of admission to [an] acute psychiatric ward.*”
“*In general, individuals have the right to refuse participation. In some situations, the answer is more difficult; the intervention evaluated in our study was [an educational] program against drugs. It was considered part of the normal curriculum, although experimental. In this case, the right to refuse the intervention [was] hardly applicable, [because it was] not applicable to the other normal lessons of the school curriculum.*”
**Need for international ethical guidelines**
“*We need direction on ethical challenges in cluster randomized trials to guide the ethically appropriate design and conduct of cluster randomized trials. We believe also that ethical implications of randomizing clusters/groups rather than individuals are not addressed in current research ethics guidelines.*”
“*The current rules and practices in Finland do not know how to handle cluster RCTs.*”
“*An effort to classify different alternatives with examples and comments regarding ethical aspects for each one would be very useful for IRBs that are not used to dealing with cluster trials. For researchers, it is usually a nightmare to find out that you have to deal with a non experienced IRB*”

The author survey illustrates discrepancies between author response and reporting. Thus, 4 authors responded that no ethics committee was contacted, but only one reported this in the paper; 13 authors responded that no participant consent was required, but only 4 reported this in the paper. Otherwise, among 6 of 113 reports stating that no consent was required (Table 2), for 2 of them, the authors did not confirm this point in the author survey.

Because CRTs are prone to selection bias, authors must fully describe how participants are recruited and the nature of the information provided (i.e., whether the nature of the intervention/group was provided to participants). However, we found a major shift between author responses and reporting. For instance, 44 authors asserted that recruitment took place before clusters were randomized and for 8 more that the trial involved blinded independent recruiters, whereas this information was given in only 9 (20.5%) and 1 (12.5%) of the associated reports. Finally, 31 authors responded that the group allocation was not specified for participants, but only 4 (12.9%) reported this in the paper.

### Authors' opinion of the management of participant informed consent

Table 6 displays some authors' comments about the management of participant informed consent. All concepts previously discussed were stated: existence of different levels of informed consent, type of consent (i.e., consent for intervention vs. consent for collection of data), partial information given to participants, and finally, the ability to avoid the assessed intervention. Finally, several authors called for international ethical guidelines.

## Discussion

Among the 173 reports of CRTs we reviewed, 23.7% lacked at least one piece of information regarding ethics committee approval and participant consent. According to corresponding author answers (113 answers, 65.3% response rate), only 33.9% of the trials had ethics committee approval, participant consent, and consent for administration of an intervention. Otherwise, for more than half of the trials, the participant consent was limited to data collection only. Finally, we estimated that only 56.6% of the 113 CRTs were free of potential selection bias.

To our knowledge, the present study is the second to address this issue, the other is the Taljaard et al. study [23]. The two studies, although concomitant, were independent. Moreover, the present study included a survey of corresponding authors, to complete the information reported in the manuscript, for a better appraisal of the very nature of the information provided to participants and the risk of selection bias.

### Reporting on ethics committee approval and participant informed consent

We show better reporting of ethics committee approval and participant informed consent than Taljaard *et al* [23], although still not satisfactory. The discrepancy may be explained by the study period: we selected reports published in 2008, whereas Taljaard *et al* considered a period between 2000 and 2008 and found better reporting over time, with a 95.0% rate of reporting for both ethics review and participant consent in published findings of a 2008 sub-sample.

### What do participants consent to?

As explained by Hutton [14], participants of CRTs may actually consent to both administration of an intervention and collection of data or collection of data only. In the latter case, we may also distinguish whether data are routinely collected or whether additional examinations are needed. None of the studied reports provided such a degree of specification, but more than half of the authors who answered this question acknowledged that participants consented to collection of data only. The nature of participant informed consent is intrinsically linked to the nature of the intervention assessed: some interventions imply no opt-out option [25]. The institutional review board must be aware of this nature of CRTs, and trialists should report this level of specification.

### Participant information and methodological issues

In clinical research, participant informed consent remains the cornerstone of ethical requirements. However, as asserted by Donner *et al* [1], “*ethical guidelines for randomized trials have been written with individually randomized trials in mind and are, consequently, only partially applicable to [CRTs]*”. The conceptual difference is that in most CRTs, participant inclusion (and therefore collection of informed consent) occurs after cluster randomization (recalling the Zelen design), and therefore, ethical issues have methodological consequences. Complete participant information after cluster randomization is a source of selection bias [18], [19] and may compromise the internal validity of the trial because allocation concealment is questioned. Moreover, providing complete information to any participant can induce contamination, whereas cluster randomization aims to prevent such contamination [13], [15], [20]. Therefore, we may face an ambivalent situation in providing complete information: participants may fully control whether or not they are enrolled in the study, but the trial is methodologically unsound, which raises ethical concerns because of lack of scientific validity [25], [26], [27].

### Reporting the participant selection process

Reporting the participant selection process is of poor quality in reports of CRTs. Only a minority of reports explicitly stated the chronology of recruitment, namely, whether participants were recruited before cluster randomization and whether the recruiter was blinded to cluster allocation [24].

As well, few reports specified whether the information given was complete or differential, and this lack of reporting prevented the reader from appraising the risk of selection bias.

### Need for international ethical guidelines

Some authors acknowledged that they encountered difficulties with their ethics committees, specifically in justifying not asking for informed consent. Authors also called for international ethical guidelines, which will soon be the case, thanks to the Taljaard *et al* program [28], [29]. Of note, establishing these guidelines calls for “prudence”: participant protection is not debatable, but we should not lapse into excess. Such a point was well summarized by one of the authors, a psychiatrist:

“*There is a double standard – many cluster trials are assessing what happens if you intervene at a group level in terms of service provision or public health. But many interventions are introduced into routine practice without the rigours of a cluster trial and far fewer safeguards to patients involved. Yet because it's a change in “routine” practice, no one considers the ethics of introducing a change whose consequences are not understood. I think this is ethically dangerous. However, if researchers make genuine efforts to study planned changes to systems, ethics becomes a big issue… This [situation] can then be a barrier to the assessment of interventions in clinical or public health settings, which could actually cause harm, or may be a waste of money. It is ethically much preferable to conduct the research acknowledging that sometimes consent cannot be complete than to impose a change on a system with no consent and no information on potential harms to the population involved.*”

This point has also been raised recently in the more general context of the Regulations Governing Research with human subjects [30] and a proposal for a new category of research risk: D*e Minimis* risk [27]. Rhodes *et al* asserted that “*bioethicists and [institutional review board] members often seem to give insufficient weight to the importance of social benefit*” and consider that “*obtaining informed consent should not be an absolute requirement for studies that involve only this subcategory, vanishingly small level of De minimis risk*.” As examples, the authors thus recommended considering studies aimed at tracking infectious disease or adopting new methods of decontamination in a hospital (which are often planned as CRTs) as not requiring informed consent.

### Study limitations

Our study has some limitations. First, the methods given in published reports may differ from the real trial methods [31], [32]. Second, although our survey response rate of 65.3% can be considered high, we lack information from nonresponders, who, we have shown, conducted trials slightly different from those conducted by responders in terms of settings and nature of the intervention. Moreover, for published reports, the corresponding authors' answers to the survey may differ from the real trial situation. Third, we focused on participant information and consent but did not investigate participant safety issues, which is also of ethical concern. However, safety issues are very specific in cluster randomized trials (e.g., if the cluster unit is a geographical area or if the intervention involves implementing new guidelines with outcomes collected from databases), and this point was beyond the scope of the present paper, although of great interest. Fourth, we classified trials at risk of selection bias that exhibited active participant recruitment with incomplete allocation concealment. However, the risk of selection bias may differ by studied intervention, which we did not quantify. Fifth, the impact of partial or differential information on participant recruitment may also differ by studied intervention, which we did not quantify, nor we surveyed authors about this point. Finally, although we collected some opinions from the authors of the reviewed cluster randomized trials, the present study was not conducted as a qualitative study for better understanding the reasons for submitting to ethics committees or not or obtaining participant consent or not.

### Implications for future CRTs

Participant informed consent perhaps should be handled differently in CRTs than in individually randomized trials because of methodological consequences. Ethics committee approval must be obtained. However, these ethics committees should scrutinize the methodologic aspects of the protocols for a better appraisal of potential selection bias. If individual consent is required, we invite trialists to specify the nature of the consent, in agreement with the 3 types defined by Hutton [14]. Finally, trialists should improve their reporting of the participant selection process and the nature of the information provided to participants of each group.
